# Bridging the gap: strategies for recognizing and managing post-COVID conditions

**DOI:** 10.3389/fmed.2024.1395420

**Published:** 2024-11-19

**Authors:** Muhammad H. Khan, Richard C. Becker

**Affiliations:** ^1^Division of Internal Medicine, University of Cincinnati Medical Center, Cincinnati, OH, United States; ^2^Division of Cardiovascular Health and Disease, University of Cincinnati Medical Center, Cincinnati, OH, United States

**Keywords:** post-COVID condition, long COVID, COVID-19, primary care, screening tools, assessment tools, guidelines

## Abstract

Post-COVID conditions (PCC), pose significant challenges for healthcare providers, employers, community leaders, and patients because of their wide-ranging, fluctuating, or persisting symptoms without well-established diagnostic tests to secure a diagnosis. Estimates suggest that up to 20–30% of adults recovering from COVID-19 develop PCC, potentially affecting millions or tens of millions of people in the United States alone. The ongoing endemic coupled with the prevalence of PCC underscores an urgent need for awareness and an understanding of potentially associated conditions, long-term management strategies, and cost-effective screening approaches for cardiovascular health. Individuals experiencing PCC present with a wide array of symptoms. Prevalence of chronic conditions such as post-infectious pulmonary fibrosis, cardiomyopathy, and accelerated coronary artery, cerebrovascular, and peripheral vascular disease further emphasizes the need for community-wide policies and practices. Screening for PCC is recommended, particularly among high-risk individuals, including those with comorbid conditions and exposure to specific SARS-CoV-2 variants, to facilitate early diagnosis, counseling, targeted interventions, and improved quality of life. The aim of this review is to highlight the urgent need for awareness, understanding and management of PCC, focusing on development of screening strategies and targeted interventions to help facilitate early diagnosis and enhance the quality of life for affected individuals. In our view early detection and management of PCC not only improves the quality of life but also improves psychosocial health. Patient-centered approaches, standardized screening tools, and initiatives aimed at enhancing understanding and treatment options, such as the RECOVER and N3C programs, are essential for effective management of PCC.

## Background

Post-COVID conditions (PCC), also known as post-acute sequelae of SARS-CoV-2 infection (PASC) or Long COVID, encompass a wide range of new, returning, or ongoing symptoms that present for ≥4 weeks after infection with the virus. The Centers for Disease Control and Prevention (CDC) estimates that 20–30% of adults who contract COVID-19 also develop PCC, potentially impacting up to 30 million adults in the United States (1). As of August 2024, United States CDC COVID Data Tracker^1^ reports the weekly COVID-19 incidence of over 10,000 cases with over 1000 weekly deaths. In the past 6–9 months, weekly COVID-19 new-case hospitalizations and deaths were estimated at 15,000 and 1,200, respectively. Accordingly, COVID-19 persists as an endemic illness with a large number of people being exposed to the risk of developing PCC, especially in densely populated areas.

Recognizing the substantial burden of PCC, the National Center for Health Statistics, in partnership with the United States Census Bureau, developed a 20-min experimental Household Pulse Survey to assess the prevalence of PCC (2). According to this survey, the percentage of individuals with PCC among those with COVID-19 ranges from 27 to 35%, aligning with CDC estimates. Acknowledging the significant impact of PCC on health, the Office of Civil Rights has issued guidelines detailing the criteria under which PCC may be recognized as a disability in accordance with the American with Disabilities Act (3).

This development underscores the urgent need to acknowledge and address the concerns of patients with PCC. In this context, we aim to focus on equipping primary providers with practical strategies for early recognition and management of PCC, a critical aspect that has been underrepresented in current literature. We also highlight the urgent need for awareness and understanding of PCC, by emphasizing on development of effective screening and assessment tools that can accurately diagnose PCC, while also ensuring the implementation of appropriate interventions and policies.

## Symptoms of PCC

According to a widely cited meta-analysis, individuals with PCC may present with 50 different signs and symptoms of varying severity (4). A recent prospective observational cohort study found that a significant proportion of patients infected with SARS-CoV-2 experienced a diverse range of symptoms. This study revealed that the adjusted odds ratios (aOR) for 37 different symptoms were 1.5-fold or higher in individuals infected with SARS-CoV-2. Among these, the most commonly reported near-term symptoms contributing to PCC include post-exertional malaise (aOR of 5.2), brain fog (aOR of 4.5), dizziness (aOR of 3.4), fatigue (aOR of 2.9), and gastrointestinal issues (aOR of 2.7) (5). Furthermore, other recently published studies have reported the incidence rate of fatigue among COVID-19 patients is approximately 10.2 per 100 person-years with a hazard ratio of 1.68 when compared to non-COVID-19 control group (6). The PRIME post-COVID study revealed significant prevalence of post-exertional malaise and orthostatic intolerance in individuals with PCC, with 48.1% of women and 41.2% of men experiencing post-exertional malaise, and 27.9% of men and 29.3% of women reporting orthostatic intolerance (7). Employing lexical analysis can be instrumental in monitoring the prevalence of PCC symptoms, as shown in a recently published study that identified evolving patterns of musculoskeletal symptoms of PCC over several phases of the pandemic (8).

In the long term, patients with chronic conditions such as coronary artery disease or chronic lung disease have an increased risk of accelerated or worsening symptoms. Emerging evidence has shown that post-COVID-19 pulmonary fibrosis, a complication of COVID-19 pneumonia, is observed in approximately 7% of patients across multiple studies, underscoring the substantial impact of COVID-19 on long-term respiratory health (9). Furthermore, COVID-19 has been associated with an increased risk of acute cardiovascular and cerebrovascular events owing to its direct endothelial, immune, and thrombotic effects. A systematic review and meta-analysis that included studies with over 20 million individuals, found an increased risk of myocardial infarction in patients following COVID-19 compared to control groups. These findings emphasize the importance of recognizing COVID-19 as a potential risk factor for future cardiovascular and respiratory complications within the patient population and an urgent need for developing a focused algorithm for diagnostic testing as discussed below (10).

## Who should be screened?

Screening for PCC is recommended for patients at high risk. A recent meta-analysis identified several high-risk characteristics, including female sex (OR, 1.56), age over 40 years, higher body mass index (body mass index ≥30), current smoking status, and various comorbidities, such as asthma, chronic obstructive pulmonary disease, diabetes, ischemic heart disease, and immunosuppression (11). Per the CDC, racial and ethnic minority groups, individuals with pre-existing substance use disorders, the homeless, and those in correctional facilities have experienced a greater impact from COVID-19. This has resulted in a higher incidence of post-COVID Conditions (PCC). Additionally, these groups often have less access to primary healthcare and are disproportionately affected by chronic conditions that increase their risk of developing PCC (1). Moreover, evidence indicates that different SARS-CoV-2 variants as well as timing and duration of treatment intervention can also affect the incidence and severity of PCC. A longitudinal cohort study of over 8,000 adults with confirmed COVID-19 revealed that the Alpha and Delta variants were associated with a higher likelihood of developing PCC than the Omicron variant or subvariants (12). The findings of several studies indicate that initiating treatment earlier is associated with a reduced risk of developing PCC, compared to that associated with delayed treatment interventions (13). The protective effect of mRNA vaccination against PCC has been demonstrated in several studies, showing that vaccinated individuals are less likely to develop PCC (12). Recognizing the characteristics of high-risk populations, including vaccination status and the predominant circulating variant of the virus, is essential. Given the high volume of patients, we suggest a targeted approach that will make screening more feasible. We recommend prioritizing patients who exhibit at least one high-risk characteristic, present with at least one persistent symptom, are unvaccinated with an mRNA vaccine, and who have contracted COVID-19 with the Alpha or Delta variant.

## Why should screening be undertaken?

Screening and identifying individuals at risk of developing PCC offers several benefits. The early detection of at-risk individuals facilitates timely antiviral treatment intervention and symptom management, potentially reducing the severity and duration of PCC symptoms (13). Early identification not only translates to improved patient outcomes in both the short and long term but also helps mitigate the impact of PCC on individuals’ quality of life and productivity, including the potential loss of work years.

The use of Household Pulse Survey as a screening tool has provided valuable insight into the impact of PCC and enabling healthcare providers to recommend appropriate therapies. Data utilizing this survey is recorded by the US Census Bureau which suggests approximately 14 percent of population who responded to the survey experienced PCC. This data also shows the association of long COVID with negative affect, physical mobility issues, and mental health problems as per a recent study (14). Currently, data from the U.S. Census Bureau has not been specifically analyzed to assess the impact of this screening tool on patients who were screened and subsequently referred for treatment. As a result, the clinical implications and patient outcomes associated with the Household Pulse Survey remain undetermined. Sharing data from the Household Pulse Survey with government bodies such as the Centers for Disease Control and Prevention and the Department of Health and Human Services can provide timely insights into the incidence, prevalence, and geographical variation of long COVID, as well as its broader impact on health and well-being.

A significant financial impact has been documented among persons with PCC. For instance, The COVID-19 Longhauler Advocacy Project conducted a survey last updated in January 2022, estimated approximately $88 billion in lost income due to PCC. This survey also found that at least 44% of individuals with PCC were unable to work, and 51% had to reduce their work hours due to the deteriorating quality of life (15). Another study, focusing on individuals under 65 years of age enrolled in commercial health plans or self-insured employer groups in the United States, assessed the additional medical costs associated with PCC. This study found that over a 6- month period, children with PCC incurred an average additional medical cost of $ 1,011 each, while for adults it was $ 1,562. These increased costs were extrapolated to the entire cohort, estimating an additional economic burden of up to $47 million for children and $ 308 million for adults with PCC over the study period (16). Understanding the economic impact of PCC is necessary for the implementation of community-wide policies that address the financial and social burden of this condition.

## Where and how to screen for PCC

Screening for PCC can be conducted in a variety of settings: ambulatory clinics, citywide health fairs, community gathering sites (such as places of worship, barber shops, hair salons, and fitness centers), through social media platforms, using wearable fitness apps, and in educational and workplace environments (17, 18). A focused and brief questionnaire that is easy to follow can assist with identifying individuals at risk of PCC. These individuals can then consult their primary care providers and complete a 5-question screening survey, as employed by the National Center for Health Statistics in its Household Pulse Survey. Primary care providers are equipped to interpret the survey results and determine whether further investigation is required (Table 1). Importantly, educating primary care providers enables them to recognize potential biases in self-reported data from such surveys, allowing them to serve as a checkpoint prior to initiating further testing.

**Table 1:** Screening survey for identifying cases of PCC.

Screening questions from household pulse survey	
Question	Answer choices
1. Have you ever tested positive for COVID-19?	Yes or No
2. How would you describe your coronavirus symptoms?	I had no symptoms; I had mild symptoms; I had moderate symptoms; I had severe symptoms
3. Did you have any symptoms lasting 3 months or longer after COVID-19?	Yes or No
4. Do you have symptoms now?	Yes or No
5. Do these long-term symptoms* reduce your ability to carry out day-to-day activities compared with before COVID-19?	Yes, a lot; Yes, a little; Not at all

*Long term symptoms may include tiredness or fatigue, difficulty thinking and concentrating, forgetfulness, or memory problems (sometimes referred to as “brain fog”), difficulty breathing or shortness of breath, joint or muscle pain, fast-beating or pounding heart (also known as heart palpitations), chest pain, dizziness on standing, menstrual changes, changes to taste/smell, or inability to exercise.

Source: National Center for Health Statistics. U.S. Census Bureau, Household Pulse Survey, 2022–2023. Long COVID. Generated interactively: from https://www.cdc.gov/nchs/covid19/pulse/long-covid.htm

For patients identified as potentially benefiting from additional testing, the CDC recommends a basic panel of laboratory diagnostic tests (1). This panel includes a complete blood count with iron studies, renal function test including electrolytes, liver function test, thyroid function assessment, inflammatory markers (such as C-reactive protein, erythrocyte sedimentation rate, and ferritin), and vitamin levels (such as vitamin D and vitamin B12). The CDC also offers guidance for the use of several assessment tools and functional tests to evaluate persons with PCC (Tables 2, 3) (1). While self-reported surveys like the Household Pulse Survey are simple and user-friendly, offering a fairly accurate assessment of symptom severity and duration, they do have significant limitations. These include a lack of specificity in questions, especially those related to demographic and socioeconomic factors, potential biases such as recall bias, and the absence of a definitive temporal link to a COVID-19 diagnosis. In contrast, the assessment tools and functional tests mentioned have been extensively studied and are widely used in both clinical practice and research. Over many years, they have proven to provide reliable and validated results (19–21).

**Table 2:** Selected assessment tools available for patients with PCC, given specific symptoms and presentations.

Selected assessment tools for evaluating people with PCC	
Functional status or QoL	Patient-Reported Outcomes Measurement Information System (PROMIS) Post-Covid-19 Functional Status Scale EuroQ0l-5D
Respiratory conditions	Modified Medical Research Council (mMRC) Dyspnea Scale
Neurologic conditions	Montreal Cognitive Assessment (MoCA) Mini Mental Status Exam (MMSE) etc.
Psychiatric conditions	Generalized Anxiety Disorder-7 (GAD-7) Patient Health Questionnaire-9 (PHQ-9) etc.

**Table 3:** Selected functional tests available for patients with PCC, given specific symptoms and presentations.

Selected functional and other testing tools for people with PCC	
Exercise capacity	1-min sit-to-stand test 2-min step test 6-min walk test 10-min walk test (10MWT)
Balance and fall risk	BERG balance scale Tinetti gait and balance assessment tool
Other	Tilt-table testing Orthostatic HR assessment

Prior to undertaking these assessments for screening, it is important to note that screening for PCC presents a challenge due to lack of specific biomarkers for diagnosis and absence of standardized treatments. Therefore, screening in this context is primarily aimed at identifying individuals who may benefit from closer monitoring, supportive care and symptoms management.

To minimize the potential financial burden of testing, we recommend a patient-centered approach. To achieve this, it is essential to differentiate PCC from other common conditions like chronic fatigue syndrome/myalgic encephalomyelitis, fibromyalgia, and either sleep and mood disorders to assure diagnostic accuracy and appropriate treatment and follow-up. Specialized diagnostic tests should be reserved for persons with symptoms and findings suggestive of specific conditions, as determined by patient history and physical examination results. Further testing and consultation with specialists or subspecialists should be based on initial diagnostic findings and may include procedures such as chest radiography, pulmonary function tests, electrocardiography, or echocardiography, particularly for patients with ongoing pulmonary and cardiovascular symptoms. It has been established that clinical guidelines, which provide a framework for evaluating and managing organ-specific symptoms and thereby preventing unnecessary testing, are essential (1). The choice of testing modality should be tailored to each patient, considering factors such as sex, age, medical and social history, prior imaging, and associated costs. Healthcare professionals are advised to prioritize a comprehensive management plan that sets realistic goals, involves patients in the decision-making process and utilizes the best available evidence.

Patient symptoms following COVID-19 often remain unexplained by objective findings, highlighting the complexity and evolving nature of PCC. Healthcare professionals should be advised to communicate this uncertainty to patients, emphasizing the importance of ongoing reassessment of their progress and treatment objectives. Given the significant number of individuals affected and the wide range of symptoms associated with PCC, we advocate for the adoption of multimodal surveillance strategies. These strategies should encompass cross-sectional health surveys and prospective cohort studies, alongside the monitoring of electronic health records and the implementation of sentinel surveillance within closed clinical networks. This comprehensive approach will facilitate a better understanding of the natural history of COVID-19 and measure the impact of PCC across diverse populations and communities.

## The optimal care for patients with PCC

While PCC is recognized by organizations such as World Health Organization, CDC, Centers for Medicare & Medicaid Services, and other federal agencies, clinicians and healthcare providers must be aware of the medical, psychological, and psychosocial conditions that may have: (1) preceded COVID-19; (2) been revealed or exacerbated by COVID-19; (3) resulted from COVID-19, especially in patients with serious or critical illnesses requiring hospitalization; or (4) emerged from a combination of these factors. We advocate that PCC should not be considered a diagnosis of exclusion. Acknowledging the need for a specific diagnostic algorithm is vital, as it would greatly benefit patients, healthcare providers, insurers, and employers. Such diagnostic clarity would also help reduce stigmatization and labeling, which often unfairly affect individuals with conditions that lack clear diagnostic criteria. An approach to the comprehensive evaluation of patients with PCC is summarized in Table 4 below.

**Table 4:** A comprehensive approach to evaluation of patients with PCC.

Diagnostic testing in patients with PCC	
Step	Action
Step 1 – Initial assessment.	1. Perform a thorough past medical history, social history, family history, 10-system review of systems, and symptoms at the time of initially confirmed COVID-19, persisting or recurring symptoms, current symptoms, prior diagnostic tests, and conduct a detailed physical examination. 2. Perform vital signs (blood pressure, heart rate, and pulse oximetry) in the supine, sitting, and standing positions. 3. Perform a complete blood count, a basic metabolic panel, a thyroid function panel, and a liver function test.
Step 2 - Based on the overall findings from step 1 and a moderate-to-high clinical index of suspicion, the following diagnostic tests should be considered based on the organ system that is involved.	Cardiac testing: Electrocardiogram, Holter monitor (14-day or 30-day event monitor), Echocardiogram, cardiopulmonary exercise test. Pulmonary testing: Chest X-ray (posteroanterior, lateral), 6-min walk test, CT chest, PFT (spirometry, volumes, and DLCO) with bronchodilator. Vascular testing: Venous duplex scan, ABI, CTPA, CT angiogram aorta (ascending and transverse-thoracic aorta). Neurologic testing: CT/CTA and or MRI/MRA brain, neck and spine, tilt table test, neurocognitive testing, electroencephalogram. Rheumatological testing: ANA panel, CRP, anticardiolipin antibodies, lupus anticoagulant
Step 3- Based on the findings from tests performed in step 2, refer patient to specialist or sub-specialist.	Specialist or subspecialist evaluation, diagnostic testing, and treatment.
Step 4- Consider candidacy for ongoing clinical trials in the RECOVER initiative.	On-site research team or contact with the geographically closest clinical trial participating site.

Under the Affordable Care Act (ACA), most “Essential Health Benefits,” including the diagnosis and treatment of Post-COVID Conditions (PCC) as recommended in Table 4, are generally covered by most private insurance plans. However, the specifics of coverage, such as cost-sharing, premiums, deductibles, and out-of-pocket maximums, can vary depending on the individual plan. Federal and state programs like Medicaid and the Children’s Health Insurance Program (CHIP) provide coverage for PCC-related tests and treatments to eligible low-income adults, children, pregnant individuals, and people with disabilities. Additionally, Medicare offers coverage for PCC diagnostic and treatment services for individuals aged 65 and older and some people with disabilities, ensuring that essential healthcare services are accessible to a broad population segment (22).

Recent literature shows considerable interest in rehabilitation potential for the commonly reported symptoms of PCC including post-exertional malaise, fatigue, dyspnea and various musculoskeletal, cognitive and/or mental health impairments (23). While rehabilitation programs, particularly those with light graded aerobic exercise over 6–8 weeks, have shown improvement in lung function, physical endurance, depression symptoms and muscular strength, the effectiveness can vary significantly and in some cases patients may even experience exacerbation of symptom, underscoring the complex nature of PCC (24, 25). Additional studies endorse the benefits of healthy lifestyle practices, including physical activity and good nutrition, in improving sleep quality and overall well-being, as well as mitigating the long-term mental health impacts for those suffering from PCC (26, 27). Given lack of targeted treatment interventions for PCC, screening may seem to be of limited value. Nevertheless, screening remains crucial for the identification of individuals who may benefit from individualized care plans and supportive rehabilitation. Personalized treatment programs, tailored to each patient’s capacity, can help reduce the burden of PCC on both the health care systems and patients. A one-size-fits-all approach is ineffective and further investigation to optimize rehabilitation strategies is needed. An example of personalized care can be seen in the development of a Core Outcome Measurement Set (COMS) for long COVID, which was designed through collaboration with healthcare professionals and patients with lived experiences of the condition (28). By focusing on core outcomes like mental health, fatigue, and respiratory symptoms, clinicians can utilize these standardized tools to guide personalized care that responds directly to each patient’s needs. This practical approach ensures that care is tailored, data-driven, and evolves alongside the patient’s condition, providing a concrete method for incorporating patient-centered care in daily clinical practice.

The ongoing National Institutes of Health-led RECOVER (researching COVID to enhance recovery) initiative is focused on understanding, treating, and preventing PCC through patient-centered research, leveraging diverse participant cohorts and electronic health records to identify treatments and elucidating the mechanisms of PCC. Meanwhile, the National COVID Cohort Collaborative (N3C) initiative provides a comprehensive data platform for researchers.

## Conclusion

Optimal management of post-COVID conditions (PCC) requires recognizing it as a critical public health challenge that demands a multifaceted approach to achieve health equity. This manuscript underscores the need for targeted screening, early intervention, and tailored management plans to improve the quality of life for individuals affected by PCC. Educating primary care providers with the tools and knowledge for early diagnosis is essential, especially as PCC impacts diverse populations with a wide range of symptoms and outcomes. This editorial examines the strengths and limitations of cross-sectional screening tools like the Household Pulse Survey, which capture prevalence, demographic variations, and socioeconomic impacts of PCC, thereby providing valuable insights into the broader physical and mental health effects of COVID-19. However, longitudinal studies are needed to assess the long-term outcomes and effectiveness of early screening measures. Furthermore, implementing a patient-centered approach requires personalized care plans, such as the Core Outcome Measurement Set (COMS), to guide care based on patients’ individual needs. These tools support practical, patient-centered care that addresses core symptoms identified collaboratively by patients and clinicians.

It is essential to address the longstanding inequities affecting the physical, social, economic, and emotional health of minority groups disproportionately impacted by COVID-19 and PCC. This requires coordinated, multidisciplinary action through initiatives spanning healthcare, community support, financial assistance, housing, nutrition, and education. By advocating for patient-centered approaches, standardizing screening and assessment tools, and establishing diagnostic algorithms, we can achieve equitable understanding and management of PCC across diverse populations.

## Data Availability

The original contributions presented in the study are included in the article/supplementary material, further inquiries can be directed to the corresponding author.

## Footnotes

^1^https://covid.cdc.gov/covid-data-tracker/#datatracker-home
